# World Opioid and Substance Use Epidemic: A Latin American Perspective

**DOI:** 10.1176/appi.prcp.20180009

**Published:** 2019-01-24

**Authors:** Saul Francisco Pacurucu‐Castillo, José Marcelo Ordóñez‐Mancheno, Adrián Hernández‐Cruz, Renato D. Alarcón

**Affiliations:** ^1^ Addiction and Psychiatric Center Cuenca Ecuador; ^2^ Department of Psychiatry Cuenca University School of Medicine Cuenca Ecuador; ^3^ Department of Psychiatry Mayo Clinic College of Medicine Rochester MN; ^4^ Academic Affairs School of Medicine Universidad Peruana Cayetano Heredia Lima Perú

**Keywords:** Opioid epidemics, Latin America, cocaine, addictions, epidemiology, dual diagnosis

## Abstract

The opioid crisis is a growing social and public health phenomenon, particularly in developed countries such as the United States. Since the 1990s, this crisis has shown a variety of causal processes and consequences and has affected quality of life for millions of individuals, families, and communities across the globe. Although abuse of opioid‐based painkillers appears to have triggered the epidemic in the United States, in this article, the problem is examined with a focus on Latin America, where drug‐associated problems constitute threats to the health and quality of life for large segments of the population. After a review of the history of opium consumption and its consequences in the world and in Latin America, the authors present epidemiological data and information about regional market differences, professional involvement, clinical practices, public health realities, and prevention efforts. Recommendations are made for collaborative efforts to promote prevention policies and measures to improve the situation.

**Publisher's Note:** This article is part of a special issue titled “The Opioid Crisis: Filling in the Picture.”

The United Nations 2017 report (1) on drug abuse shows that more than 200 million people, or 4.2% of the world population, are addicted to illegal drugs. This number includes 144 million addicted to cannabis or its derivatives, 29 million to amphetamines, 14 million to cocaine, and 13.5 million to opiates (with almost 9 million addicted to heroin) (2). The annual prevalence of opioid users covers 0.6%–0.8% of the global population and consists mainly of those ages 15 to 64 years, with most consuming heroin, morphine, and nonprescription opioids. These percentages make drug abuse and addiction one of the most prevalent psychiatric disorders (3), with significant social implications (4, 5).

In recent years, many famous people (particularly in the fields of arts and entertainment) have died from opiate overdoses (6, 7, 8), placing the opioid crisis at center stage. There is now almost unanimous agreement that opioid abuse constitutes a complex public health problem (6, 9, 10). Street use of heroin mixed with fentanyl makes the situation more difficult, causing many deaths (10, 11). The opioid epidemic has been declared one of the worst national health crises in the United States, its growth having started in the 1990s (10, 12). The U.S. Surgeon General has advised against the indiscriminate prescription of opioid analgesics. Drastic control measures were announced by the U.S. Government, after reports (12) that opioid abuse leads to 1,000 visits per day to hospital emergency rooms and kills 78 people per day in the United States.

The problem looks different in Latin America (13, 14). Although the perception by health authorities and the public in Latin America is that drug abuse is a growing problem, the amount and quality of the information are lacking. There are data about the number of opioid prescriptions, but the heterogeneity of the epidemiological and diagnostic data resulting from a limited number of studies makes their reliability difficult to ascertain. Nevertheless, the purpose of this review is to approach the world opioid epidemic from a Latin America perspective, using the most appropriate available information.

## BRIEF HISTORICAL BACKGROUND

Opium is obtained and extracted from the *Papaver somniferum* poppy plant. Opioids have been used for pain control throughout the millennia by many societies and cultures. References to the medical uses of opium appeared in the first century A.D. in stories of Pliny the Elder. The Greek physician and philosopher Galen of Pergamon reported on therapeutic doses, and opioids were used by Hippocrates and Avicenna. By about the 1500s, the Portuguese introduced opium smoking in Europe, and the first reliable reports of dependence appeared in Persia and Turkey around 1515–1588 (3). By the 17th century, England had taken control of opium production and commerce across the globe. In the 1830s, morphine was purified and used as an analgesic (15).

Opium alkaloids, such as morphine and codeine, possess the same or similar properties as crude opium. Traditionally, the term *opiate* was used to describe these naturally occurring substances and the semisynthetic drugs derived from them (e.g., diamorphine/heroin), whereas the term *opioid* was used to describe the completely synthetic drugs (e.g., dextromoramide, methadone, pethidine) with similar properties. More recently, with the discovery of the opioid receptors that bind all these drugs, the term *opioid* has come to be used as the collective description of the naturally occurring alkaloids, semisynthetic derivatives, and completely synthetic drugs. Currently, the terms opiate and opioid are used interchangeably (16).

After the Chinese Empire banned the cultivation and selling of opium, England, assisted by the East India Company, established opium as a freely admitted commodity. China had 2 million opium smokers in 1850. By 1878, there were 120 million, an increase of 6,000% in 28 years (2). The opium business was established as one of the economic pillars of the British Empire. Heroin production centers were later expanded to Central and South America, southwestern Asia, Europe, and the United States. Currently, Asia is the main producer of poppy plants (opium and heroin). In Latin America, the main producers (although on a smaller scale) are Mexico, Guatemala, Colombia, and Peru, in that order. Although the Latin American population appears to be less affected by the use of heroin, the major problem in the region is the heavy narcotics traffic routed primarily to the United States (17, 18).

## The Expansion of Opioid Use

Opium has had a therapeutic use and population wide consumption for almost 4,000 years. In the 19th century, opiate dependency rose to a global scale for the first time. In Arabic medicine, opium was mostly used to treat problems, such as diarrhea. In the 15th and 16th centuries, Paracelsus considered it the “immortality stone,” and in 1660, Thomas Sydenham expanded the consumption of opium even more with the use of laudanum in medical practice.

The most relevant milestone in this historical process was the isolation of morphine from opium by Friedrich Serturner in 1803. Later, the intravenous use of morphine contributed to the most severe variety of opioid addiction. Heroin (also called diamorphine or diacetylmorphine) was developed in 1874 and promoted as a cure for morphine addiction; yet, its addictive potential was quickly identified, and its open selling was suspended in the 1920s (19).

With the British Empire's expansion, commercial routes were established between Europe and many Far East producer countries. Opium and its derivatives were commonly used in England, even among children, without medical prescription. Consumption of heroin for pleasure was popular among poets and other writers and was publicized in literary works, such as Thomas De Quincey's *Confessions of an English Opium‐Eater* (published in 1821). By the end of the 1870s, morphine addiction had expanded widely.

Opioid abuse, specifically heroin, increased in the West during the 1960s. The current crisis detonated in the late 1990s, when physicians began to prescribe increasing amounts of opioid analgesics, driven by pharmaceutical industry advertisements and increasing public demand, the latter nourished by illegal commerce. Many authors point to that period as the beginning of today's opioid epidemic (20).

## OPIOID CONSUMPTION IN LATIN AMERICA

Chinese migrants carried the practice of opium smoking to other countries by the beginning of the 17th century. In South America, a group of blue‐collar Chinese workers settled in northern Chile and established opium‐smoking dens. It has been estimated that 12%–13% of the immigrant Chinese population was engaged in this practice. Curiously, neither Chilean nor other immigrants to Chile seemed to be actively involved in the habit between 1880 and 1950. As late as the mid‐1970s, an article in the *Pan American Health Organization Bulletin* claimed that “it is possible to say that opium and its derivatives. . . are not a problem in the region,” with the exception of Puerto Rico and Panama, where heroin consumption had apparently reached high levels (21).

In countries such as Mexico and Guatemala, no cases of opium misuse had been reported by the 1990s, despite the existence of opium poppy crops in those countries (22). In turn, the National Survey on Psychoactive Drugs, conducted in Argentina in 2017 with the population ages 12–65 years, reported a lifetime prevalence of 0.1% for use of heroin, opium, and morphine (23). A somewhat similar result was obtained in Colombia (18) in 2013, where only 1.07% of the population had ever used opiates and no differences were found in use by gender. Those ages 18–34 years reported the most use. In that study, 0.14% of the respondents had used heroin at least once, and 0.03% reported use during the prior year (18).

Researchers at the San Vicente Foundation University Hospital in Medellin, Colombia, reviewed the medical history of patients diagnosed as dependent on prescribed opioids between 2011 and 2014. Of a total of 3,332 medical records, 60 patients (1.8%) met criteria for opioid dependence, although 33% of those had been misdiagnosed. Eighty‐eight percent of the sample started consumption after receiving prescribed pain medication, although the pain severity was minimal in 25% of the patients. Inappropriate use of opioids may thus have persisted because of self‐medication to relieve pain. Only 4 of 60 individuals (7%) had a cancer diagnosis. The most consumed substances were tramadol and morphine (6 of 37, 16%), oxycodone, and others; mean duration of consumption was 48 months (range 1–240 months). For one quarter of the sample, opioid use was associated with consumption of other psychotropic substances. Fifty‐five percent had started replacement treatment with methadone after years with the diagnosis of polydrug abuse, and 40% were undergoing another treatment (24).

Puerto Rico shows a proportionately higher incidence of opioid misuse compared with Latin American countries, with approximately 60,000 people with opioid misuse problems; among those, a significant number are in jail (25). These findings make the island part of the U.S. opioid epidemic, which has been triggered by the increase in legally prescribed analgesics and illegally obtained opioids.

In Brazil, access to prescription opioids is limited, so consumption there is low (26), and regional polls have shown low levels of heroin consumption. Substances which have a risk of dependence but are authorized for medical use (i.e., sedatives or tranquilizers) are subject to a governmental drug monitoring system in Brazil (17, 27). The United Nations registered a worldwide increase in the number of filled opiate prescriptions between 2009 and 2015. Codeine was the opioid most often prescribed for treatment of mild‐to‐moderate pain, but oxycodone had the largest increase in use. These patterns, together with use of drugs like fentanyl, reflect a trend toward the use of more powerful opiates (1, 28).

Health risks resulting from illicit injectable drug use include greater susceptibility to HIV, hepatitis C virus (HCV), cutaneous infections, infective endocarditis, and fatal overdoses (29). Approximately 1.6 million people in Latin America have HIV. In Argentina and Brazil, injected drug use has increased the prevalence of this infection in the last four to five decades to 50% and 48%, respectively. However, only a small group uses injectable heroin, because cocaine and its derivatives are preferred in these two countries. Hepatitis C has a prevalence among intravenous drug users of 98% in Mexico and 69% in Brazil (27, 30). The United Nations reported in 2011 that people in jail who self‐injected drugs, were likely to combine cocaine with heroin, a mix known as a speedball (29).

## Current Perspectives in Latin America

The realities of opioid use and abuse in Latin America may be deceptive if observations are limited to epidemiological findings (31). In the United Nations report mentioned above (28), although South America produced 3% of the world's morphine and heroin and <0.01% of its opium, prevalence of use is uneven. According to the Inter‐American Commission on Drug Abuse Control, consumption of heroin is low in most Latin American countries, although Colombia is the area's largest opium producer. Mexico, because of its border with the United States, has the highest incidence of use (32).

The above statistics may change rapidly for a variety of reasons. One variable is the role of the pharmaceutical industry and its marketing and expansion strategies to generate change in the medical prescription culture. In the United States, an advertising strategy that focused on cancer patients and on physicians with the highest indices of analgesic prescriptions (33) led to a 402% increase in the prescription of oxycodone, which is recommended as a less addictive alternative to morphine. After the 2017 declaration of the opioid crisis as the worst drug epidemic in the country and a national emergency in the United States, the pharmaceutical industry used the same strategies to target Latin America as its potentially strongest market (34).

The Latin American Commission on Drugs and Democracy, in its statement on the situation in the subcontinent (35), recommended focusing on drug use as a health problem, by establishing consumption‐reduction policies through information and prevention (3) and working actively toward suppressing organized crime.

## OTHER ASPECTS OF DRUG USE AND ABUSE IN LATIN AMERICA

Several features of the drug use and abuse problems that are specific to Latin America are described next.

### Market Differences

Demand for opioids varies by region because of the social and geographic characteristics of different countries. For instance, the increase in the marketing and use of opioids in Brazil can be attributed to public and professional demand for better handling of pain‐related conditions (26). Meanwhile, in Mexico, the highest concentrations of people who use injectable drugs are found in the areas that border the United States (i.e., Tijuana and Chihuahua). The HIV infection rate is 4% among people using drugs in these border zones, and more than 90% of them have also tested positive for HCV (36). Furthermore, among Latin Americans who are prescribed opioids for pain management, type and severity of side effects vary, as does effectiveness of the treatment approaches (37, 38).

Illegal opioids being produced include morphine, codeine, thebaine, hydrocodone, oxycodone, and methadone. Production has increased dramatically in recent decades. Although there are medical reasons for this increased production, they raise the risk of excessive prescribing and subsequent appearance of channels for illicit use. Buprenorphine and pentazocine have been used in many countries as substitutes for heroin, and the abuse of codeine is frequently preceded by its use in popular cough syrups (25).

### Gender

In Latin America, addiction among women is seen more negatively than addiction among men. This view results in diminished social acceptability and higher levels of rejection and public criticism for women with drug addiction, perhaps leading to less help‐seeking actions by relatives and acquaintances. The situation is undoubtedly related to sociocultural factors, family structures, community networks, and even political conditions (39).

Several prevalence studies have found males to have the highest percentage of heroin use worldwide. In Latin America, data on consumption of opioids by females older than age 14 years have shown a prevalence of 0.3% for heroin use and a prevalence of 0.1% for other drugs of abuse. It is estimated that those with a dependence disorder number 1–3 per thousand. Whereas it has been found that there is one female for every 4–5 males addicted to opiates, the ratio for demand of opioid‐assisted pain relief by males versus females is 1 to 7, respectively (31, 32, 40).

### Medical Professionals

As in other regions, physicians in Latin America have easier access than the general population and other professionals to psychoactive substances and habit‐forming drugs. A Brazilian study showed a predominance of anesthesiologists in the use of alcohol (50%), stimulants (33%), opioids (8%), and other substances (9%). Interestingly, however, the most used opioids were fentanyl and sulfentanyl, which are often prescribed for mental diagnoses, such as personality disorders (41).

Opioid analgesics are the most commonly prescribed medications and often are the first choice for management of acute and chronic pain. Added to the nonmedical use of opioids, this prescription practice increases the social and public concerns about use of these drugs (42, 43). Most problems begin with the excessive prescription of the drug by the physician, but then the patient takes the initiative to abuse the prescribed drug for its euphoric effects or as an attempt to control eventual withdrawal symptoms (37).

### Co‐Occurring Disorders

The prevalence of psychiatric disorders among opioid users ranges from 44% to 93% (44). The prevalence of these co‐occurring disorders (the so‐called “dual diagnoses” cases) throughout life among opioid‐dependent persons is 40%–80%, with depressive disorder being the most frequent, ranging from 4% to 54%. In comparison, the overall prevalence of depression ranges from 25% to 30% in the general population.

The prevalence of anxiety disorders varies in different studies and populations; phobic disorder is the most prevalent, with simple phobia at 4% and social phobia at 3%–6%. The variability of results is reflected by another study that found prevalence figures of 33% and 39% for simple and social phobia, respectively (45). Diagnoses of schizophrenia (0.1%), eating disorders (0.7%), and obsessive‐compulsive disorder (0.3%) among drug abusers are similar to those found in general populations (46).

Personality disorders frequently accompany opioid dependence, with some studies showing that 33%–66% of patients meet criteria for one or several of these diagnoses. The most frequent type in this group is antisocial personality disorder (47). Finally, there is evidence that sleep disorders (particularly sleep apnea) are more frequent in people who consume opioids; sleep disorders may include poor quality of sleep as a triggering factor and abnormalities in the architecture of sleep as a pathogenic mechanism (48).

### Legal Acceptance

Many Latin American countries have established legally acceptable amounts of opioid possession, which vary by country. In Mexico, the General Health Law and Federal Penal Code, popularly known as the *Narcomenudeo* Reform, decriminalized possession of small amounts of drugs and stipulated for personal use a maximum of 2 g of opium, 50 mg of heroin, 5 g of marijuana, 500 mg of cocaine, 40 mg of methamphetamine, and 0.015 mg of lysergic acid diethylamide (LSD) (36, 38).

In contrast, in Puerto Rico, possession of even a small bag of heroin can lead to prosecution and punishment rather than treatment or rehabilitation (25). In Ecuador, portability is a judicial instrument rather than an option accepted by the health system. There have been large and continuous controversies and subsequent changes to its terms (49, 50). The legal possession limit in Ecuador is 10 g for marijuana or hashish; 4 g for opium; 100 mg for heroin; 5 g for cocaine; 0.020 mg for LSD; and 80 mg in powdered, granular, or crystal methylenedioxyamphetamine (MDA) or methamphetamine or pieces weighing ≤ 400 mg.

Brazil partially decriminalized the possession of certain drugs and doses and succeeded in replacing prison sentences with educational measures and community service. In Chile, a special authorization for the cultivation of cannabis is required, whereas Colombia, like Ecuador, adds the preferential use of a portability table. In 2013, Uruguay approved a law regulating the sale of marijuana (18, 21, 26, 45).

### Public Health Issues

Pain is undoubtedly the main referent between legal and illegal use of opioid medications, and cancer is the clinical condition most amenable to their use. Sixty to 80% of pain symptoms of patients with cancer are attributed to the condition itself, 20%–25% to the anticancer treatments, and 5%–10% to causes other than cancer (40, 42, 45). In turn, emotional and cognitive factors increase the persistence and severity of pain. Whereas some physicians resort quickly to the use of opioids for pain patients, some patients refuse to take such compounds, generating an interesting dilemma. Studies have shown that 40%–70% of patients fail to manage their pain adequately because of “opium phobia” (45). For example, a study in Sao Paulo, Brazil, showed that 19.2% of patients resisted using the morphine prescribed by their doctors, with the main reasons being fear of dependency (53.4%), religious principles (25%), dependency phobia (65.2%), fear of adverse effects (34.7%), and fear of developing tolerance (30.4%). Most of the study participants related morphine to worsening health (67.9%) and as a factor that put them closer to death (41.1%).

### Prevention

Opioid replacement therapy has many benefits; approximately 85% of the United Nations member countries reported a low‐to‐average proportion of participants in these programs (i.e., less than 40% of opium‐dependent persons being covered), while more than 5% of countries reported that they did not have any kind of coverage (47).

Harm reduction, also known as mitigation, programs have had little presence in Latin America, except in Brazil and Argentina where, in addition to methadone programs, syringe distribution campaigns to prevent the spread of HIV have been implemented (1, 28). Mexico seems to be the only country in the region that has a comprehensive program for alternative treatments and exchange of needles and syringes (36, 38). Argentina, Brazil, Paraguay, and Uruguay have programs for the administration and management of new synthetic psychoactive substances. Moreover, to avoid an epidemic such as the one in North America, Brazil has introduced methods for prescription and control of opioid medications, allowing patients to access risk prevention services and programs (42).

## DISCUSSION

There are a variety of routes through which substance abuse in general, and opioid abuse in particular, can materialize. Researchers of the current opioid crisis, government officials, international agencies, and community leaders concur in pointing to the attempt to effectively treat pain as a common, growing factor in this process (27). The subsequent increase in prescriptions of opioids came without consideration of the risks of addiction: the fact is that the number of people addicted has increased across social sectors, age groups, genders, and acute and chronic medical and psychological conditions (27, 35). The consequences of a significantly increased number of users (particularly in developed countries) include intensification of traffic in narcotic drugs, emergence of black markets, earlier age of onset of abuse and dependence, and greater prevalence of overdose and subsequent death (37).

Overall, increased disability reduces quality of life for families, social groups and, sometimes, entire communities. Opioid addiction is already considered a public health problem in many latitudes. Developed countries consume 79% of the world's morphine. Although 80% of the world's population is located in developing countries where most of the drug‐producing plants are grown, consumption in these areas has stayed at a relatively low 6% (37, 47).

A broad look at opioid use across the world and in Latin America leaves no doubt that health professionals as well as policymakers should make prevention the main pillar of any long‐term approach to the opioid crisis and to substance abuse in general. Such a perspective must inspire relevant legislation, public and professional education and training programs, well‐qualified assistance agencies, and well‐funded, pragmatic research initiatives. Such approaches also require an objective assessment of short‐ and long‐term accomplishments.

It is equally valuable to consider therapeutic alternatives for the management of opioids as medications and as drugs of abuse. Addictions must be thoroughly evaluated by competent, multidisciplinary teams to obtain a comprehensive clinical assessment of all the etiopathogenic factors involved.

Treatment processes and their results must be assessed. Complete care requires close monitoring of sociocultural, family, environmental, and clinical factors of patients with complex (and often comorbid) psychiatric disorders. Finally, policies that ensure safe access to and safe medical use of opioids are much needed.

The availability and use of opioid analgesic agents remain low in many countries of Latin America, a situation that is explained by factors as varied and heterogeneous as poverty, religious precepts, family cohesiveness and sociocentrism, and even the dominant prevalence of alcoholism (51, 52).

Additionally, some Latin American countries have taken the lead in establishing measures aimed at protecting against negative factors that aggravate drug use and its consequences. When a study in three Colombian cities showed that 17.5% of users of injectable heroin had HCV, the initiative to make syringes available in pharmacies or shops gained ground, and harm‐reduction policies also have been implemented in Argentina and Brazil (26, 30, 38). In Mexico, the use of remifentanil, a 4‐anilidopiperidine derivative of fentanyl, a potent, ultra‐short‐acting, synthetic µ‐opioid receptor agonist, is considered a safe analgesic alternative in obstetrics (53), although this has not been definitively proven.

A panel of experts called by Change Pain Latin America, a regional branch of an international nongovernmental organization, gathered in Guatemala to discuss the most frequently cited factor in the growth of narcotic analgesics and opioid use (54). The group recognized that opioids are more effective than other drugs at reducing pain and pointed to major limitations in daily life activities as their main indication. The group also made clear, however, that the health professional must be well acquainted with the patient and his or her problems and with the risks of the medications in order to establish a comprehensive management program with a reasonable trial period and close and permanent monitoring.

## CONCLUSIONS

Consumption of illegal drugs, particularly opium derivatives, has many centuries of practice and has resulted in myriads of epidemics throughout human history. Any attempt to counteract the current situation must combine strong policies; education campaigns; professional alertness; and the joint work of health authorities, medical professionals, social leaders, and pharmaceutical companies. It is crucial to establish or strengthen appropriate policies for providing information on and producing, importing, and marketing opioids. With pain management as a triggering factor for the increase in opioid use, prescription practices, therapeutic protocols, advertising styles, and regulatory instruments must constitute a harmonious as well as effective armamentarium for pain control efforts.

Latin American countries must remain vigilant of developments in other parts of the world and adopt measures to maintain or even reduce their current low levels of opioid consumption, while fighting pervasive use of alcohol and other drugs (e.g., cocaine, hallucinogenic agents, medicinal herbs) that threaten public health. Leaders of medical schools and other schools of health professionals should continue to update their curricula on this subject, while pursuing and performing relevant research in related areas.
